# Effectiveness of Amikacin liposome inhalation suspension for refractory *Mycobacterium avium* complex pulmonary disease at 6 months post initiation

**DOI:** 10.1186/s12890-024-03261-w

**Published:** 2024-09-10

**Authors:** Naohisa Urabe, Susumu Sakamoto, Nozomi Tokita, Hiromichi Yoshida, Yusuke Usui, Hiroshige Shimizu, Muneyuki Sekiya, Shion Miyoshi, Yasuhiko Nakamura, Kazutoshi Isobe, Kazuma Kishi

**Affiliations:** Department of Respiratory Medicine Omori Medical Center, Toho University, 6-11-1 Omori-Nishi, Ota-Ku, Tokyo, 143-8541 Japan

**Keywords:** *Nontuberculous mycobacterial* pulmonary disease; *Mycobacterium avium* complex; Amikacin liposome inhalation suspension

## Abstract

**Background:**

Amikacin  liposome inhalation suspension (ALIS) improved sputum culture conversion rate at 6 months for patients with refractory *Mycobacterium avium* complex pulmonary disease (MAC-PD) in an international phase 3 trial. Patient characteristics and chest high-resolution CT (HRCT) findings associated with ALIS effectiveness are poorly documented.

**Objective:**

We aimed to clarify ALIS effectiveness for refractory MAC-PD at 6 months, elucidating associated patient characteristics and chest CT findings.

**Methods:**

We reviewed medical records of 12 patients with refractory MAC-PD for whom ALIS treatment was initiated at Toho University Omori Medical Center from November 2021 through September 2022. All patients demonstrated treatment persistence for at least 3 months. They were divided into culture conversion and non-conversion groups using sputum culture conversion status after 6-month ALIS treatment initiation. Clinical and radiological characteristics were compared.

**Results:**

Seven of the 12 patients (58.3%) achieved sputum culture conversion within 6 months. The culture conversion group had shorter pre-ALIS initiation treatment duration [21 months (16–25) vs. 62 months (32–69); *p* = 0.045]; lower cavitary lesion incidence on HRCT (28.6% vs. 100%; *p* = 0.028); and fewer clarithromycin (CLA)-resistant strains [0/7 (0%) vs. 3/5 (60%); *p* = 0.045]. Chest HRCT findings improved in 4 of 7 (57.1%) and 1 of 5 (20%) patients in the culture conversion and non-conversion groups, respectively.

**Conclusion:**

ALIS facilitated sputum culture conversion within 6 months in 58.3% of patients with refractory MAC-PD. Sputum culture conversion was significantly more frequent for CLA-susceptible strains and patients with fewer cavitary lesions. Improved CT findings after ALIS did not always correspond to sputum culture conversion.

## Background

The current American Thoracic Society/European Respiratory Society/European Society of Clinical Microbiology/Infectious Disease Society of America (ATS/ERS/ESCMID/IDSA) guidelines recommend a 3-drug combination therapy, including a macrolide, for patients with macrolide-susceptible *Mycobacterium avium* complex pulmonary disease (MAC-PD) [1]. The success rate of recommended guideline-based therapy (GBT) is unsatisfactory. A systematic review reported 61.4% of MAC-PD patients achieved sputum culture conversion with GBT [2]. A 2020 guideline recommended addition of Amikacin liposome inhalation suspension (ALIS) to treatment regimens for patients with refractory MAC-PD who do not achieve sputum culture conversion after at least 6 months of GBT [1]. ALIS was developed to achieve high trough concentrations of Amikacin (AMK) in peripheral lung tissue and alveolar macrophages with minimal systemic adverse effects. ALIS contributed to the primary endpoint of improving sputum culture conversion rate at up to 6 months in the international phase 3 open label CONVERT trial [3]. However, the characteristics of patients and chest CT findings associated with effectiveness of ALIS are poorly documented. Following approval in Japan (March 2021), we administered ALIS to 12 patients with refractory MAC-PD. This study was designed to clarify patient characteristics and elucidate chest CT findings typically associated with effectiveness of ALIS treatment.

## Methods

### Study design

This single-center retrospective cohort study enrolled 12 patients with refractory MAC-PD treated with ALIS. They had no therapeutic response to prior GBT, thus ALIS treatment was initiated at Toho University Omori Medical Center from November 2021 through September 2022. Each patient satisfied ATS/ERS/ESCMID/IDSA guidelines diagnostic criteria for MAC-PD. We evaluated the proportion of sputum culture conversion and safety of ALIS at 6 months.

Based on sputum culture conversion results at 6 months after ALIS treatment initiation, the 12 patients were divided into a culture conversion group and a non-conversion group. Clinical and radiological characteristics were then compared. Additionally, chest CT score, Chronic Obstructive Pulmonary Disease (COPD) assessment testing (CAT) score [4], and body weight at 6 months were compared with baseline values for both groups. Weight gain or loss was defined as a change of 1 kg or more.

### Definition of refractory MAC-PD and sputum culture conversion

Refractory MAC-PD was defined as persistent MAC detection in sputum culture after 6 months of GBT. Sputum culture conversion was considered achieved with three consecutive negative sputum cultures.

### ALIS Administration

A 590 mg-dose of ALIS (Arikayce; Insmed Inc., Bridgewater, NJ) was administered once daily by inhalation using the eFlow® rapid nebulizer system (Lamira; PARI Pharma GmbH Co., Ltd., Munich, Germany), according to the manufacturer’s instructions. Patients were hospitalized for 2 or 3 days to receive hospital-based training on proper inhaler technique. After hospital discharge, inhalation therapy was continued at a fixed time every day in principle, but the frequency of use was reduced based on adverse events.

### Data collection

We collected data on age, sex, body weight, body mass index (BMI), smoking history, serum anti-glycopeptidolipid (GPL)-core IgA titer, comorbidities, chest and paranasal sinuses CT, and sputum and bronchoscopy culture results. At our institution, all patients with MAC-PD, including those treated using ALIS, routinely undergo chest CT scans, weight measurements, and CAT score evaluation approximately 6 months after treatment initiation to assess therapeutic efficacy. The data utilized in this study were collected retrospectively from these evaluations, which were performed as part of our routine clinical practice. Baseline chest CT scans were performed within 3 months prior to ALIS installation. The 6-month follow-up chest CT scans were performed at a median of 171 (168.8–178.3) days after ALIS installation. Levels of serum IgA antibody against the GPL-core antigen of MAC were measured using an enzyme immunoassay kit (TAUNS Laboratory Inc., Shizuoka, Japan) [5].

### Microbiological Examinations

Sputum specimens were subjected to Ziehl–Neelsen and Gram staining and cultured for mycobacteria, other bacteria, and fungi. PCR assays for *M. tuberculosis*, *M. avium*, and *M. intracellulare* were also performed using DNA-DNA hybridization to identify non-MAC species. Minimum inhibitory concentration (MIC) of clarithromycin (CLA) and AMK were determined using BrothMIC NTM with 7H9 Middlebrook liquid medium (Kyokuto Pharmaceutical Industrial Co., Ltd., Tokyo, Japan). Based on the Clinical and Laboratory Standards Institute (CLSI) standard 24A, CLA resistance was defined as MIC ≥ 32 μg/mL [6]. AMK resistance was defined as MIC ≥ 128 μg/mL, which is the CLSI-recommended breakpoint for ALIS [7].

### Chest CT score

Chest high-resolution computed tomography (HRCT) score was reviewed as previously described by Kim HS et al. [8]. We evaluated for the presence and extent of parenchymal abnormalities, including bronchiectasis, cellular or inflammatory bronchiolitis, nodules of 10–30 mm in diameter, airspace consolidation, and cavities. A total score of 30 was allocated for the overall extent of a lung lesion in each patient. Chest CT scores were reviewed independently by 2 respiratory physicians with over 15 years of experience; the sum of each score divided by 2 was used for each item. Improvement in chest CT score was defined as a decrease by at least one point while worsening was defined as an increase by at least one point.

### CAT score

CAT score was specifically designed to evaluate health-related quality of life (HRQoL) in patients with COPD [9]. The usefulness of the CAT score for assessing HRQoL in patients with NTM-PD has also been reported [10], but the minimal clinically important difference (MCID) in NTM-PD has not been investigated. In this study, the MCID for the CAT score was set at 3 points per a previous study on bronchiectasis [11].

### Statistical analysis

Data are presented as number of patients and percentages. Age, BMI, chest CT score, and CAT score are expressed as median (with interquartile range). Associations of categorical and continuous variables between the two groups were tested using the Chi-squared or Fisher’s exact test, and the Mann–Whitney U test, respectively. The paired t-test was used to compare the baseline and 6-month time points for the two groups, respectively. A p-value of ≤ 0.05 was considered indicative of statistical significance. Statistical analyses were performed using SPSS software version 25 (IBM Corp., Armonk, NY).

### Ethics

The study protocol was approved by the Ethics Committee of Toho University Omori Medical Center (Approval No. M22164). All procedures were in accordance with principles of the Declaration of Helsinki for studies involving human subjects (2008). The requirement for informed consent was waived by the IRB per the retrospective study design with anonymized patient data.

## Results

### Effectiveness of ALIS at 6 months and associated clinical characteristics

Of the 12 patients in this study, 7 (58.3%) achieved sputum culture conversion at 6 months after ALIS initiation. While 1 patient discontinued treatment at 3 months after ALIS initiation due to ototoxicity, the remaining 11 showed treatment persistence for 6 months.

The culture conversion group comprised 7 patients (7 women; median age 70 years) and the non-conversion group included 5 patients (3 women; median age 64 years). Table 1 shows the characteristics of all 12 patients at baseline. Both culture conversion and non-conversion groups showed significant differences in the proportion of women [7/7(100%) vs. 3/5(60%); *p* = 0.045], subjective sputum symptoms in CAT score [2(0.5–3) vs. 4(3–4); *p* = 0.05], total chest CT score [12(9–13) vs. 15(15–17); *p* = 0.012], proportion of patients with cavitary lesions (28.6% vs. 100%; *p* = 0.028), and proportion of CLA-resistant strains [0/7 (0%) vs. 3/5 (60%); *p* = 0.045]. Although no significant differences were found, the culture-conversion group tended to have more sputum smear-negative cases at ALIS initiation and a longer time to sputum culture positivity. Table 2 shows treatment details before ALIS treatment initiation. Treatment regimens for all patients included 3 types of drugs at baseline. Pre-ALIS treatment duration was significantly shorter in the culture conversion group [21(16–25) months vs. 62(32–69) months; *p* = 0.045].

**Table 1:** Clinical characteristics of patients on ALIS treatment

Characteristic		Total	Culture conversion group	Non-conversion group	*p*-value
No. of patients		12	7	5	
Age: (years); median (range)^a^		68 (63–72)	70 (64–73)	64 (61–72)	0.688
Sex: female; n (%)		9 (75)	7 (100)	3 (60)	**0.045**
BMI: (kg/m^2^); median (range)^a^		18.4 (16.4–20.0)	19 (16.4–19.3)	17.8 (16.6–21.9)	0.785
Smoking: never; n (%)		9 (75)	6 (85.7)	3 (60)	0.523
Positive GPL-core serum IgA result; n (%)		10 (83.3)	7 (100)	3 (60)	0.152
Comorbidities: n (%)					
Rheumatoid arthritis		0 (0)	0 (0)	0 (0)	
Sinusitis		1 (8.3)	1 (14.3)	0 (0)	1.000
Malignancy		1 (8.3)	1 (14.3)	0 (0)	1.000
Underlying pulmonary disease: n (%)					
Emphysema		1 (8.3)	0 (0)	1 (20)	1.000
Interstitial pneumonia		0 (0)	0 (0)	0 (0)	
Concomitant drug; n (%)					
Corticosteroids		0 (0)	0 (0)	0 (0)	
Immunosuppressant		0 (0)	0 (0)	0 (0)	
Biopharmaceutical		0 (0)	0 (0)	0 (0)	
Infective MAC isolate; n (%)					
*M. avium*		8 (66.7)	5 (71.4)	3 (60)	1.000
*M. intracellulare*		4 (33.3)	2 (28.6)	2 (40)	1.000
Subjective symptoms (range)^a^					
CAT score		16.5 (12.8–23.8)	13 (9.5–20.5)	21 (15–28)	0.185
Cough score ^b^		3 (2–4.3)	3 (1.5–4.5)	3 (3–3)	0.812
Sputum score^b^		3 (1.8–4)	2 (0.5–3)	4 (3–4)	**0.05**
Chest CT score					
Total chest CT score; median (range)^a^		14.5 (11.8–15.3)	12 (9–13)	15 (15–17)	**0.012**
Cavitary lesion; n (%)		7 (58.3)	2 (28.6)	5 (100)	**0.028**
Radiographic pattern; n (%)					
Non-cavitary NBE type		5 (41.7)	5 (71.4)	0 (0)	**0.028**
FC type		1 (8.3)	0 (0)	1 (20)	0.417
Cavitary NBE type		6 (50)	2 (28.6)	4 (80)	0.242
Proportion of resistant strains; n (%)					
Clarithromycin		3 (25)	0 (0)	3 (60)	**0.045**
Amikacin		0 (0)	0 (0)	0 (0)	1.000
Burden of MAC at baseline					
Time to positive culture (days); median (range)^a^		30 (27.1–34.6)	32 (27.8–38.5)	28 (25.5–32)	0.194
Positive smear with Ziehl–Neelsen staining; n(%)		9 (75)	4 (57.1)	5 (100)	0.205

*ALIS* Amikacin liposome inhalation suspension, *BMI* Body Mass Index, *GPL* glycopeptidolipid, *MAC* Mycobacterium avium complex, *CAT* COPD assessment test, *CT* Computed tomography, *NBE* nodular bronchiectatic, *FC* fibrocavitary

^a^interquartile range

^b^Score for subjective symptoms of cough and sputum, included in CAT score

**Table 2:** Treatment details before initiation of ALIS

Characteristic	Total	Culture conversion group	Non-culture conversion group	*p*-value
No. of patients	12	7	5	
Treatment duration before ALIS: (months); median (range)a	25.5 (20–39.5)	21 (16–25)	62 (32–69)	**0.045**
Number of GBT drugs in regimen (at baseline)				
2 / 3 / 4 or more	0 / 9 / 3	0 / 7 / 0	0 / 2 / 3	
Drug, class (at baseline)				
Macrolide	12 (100)	7 (100)	5 (100)	
Ethambutol	10 (83.3)	6 (85.7)	4 (80)	
Rifamycin	11 (91.7)	7 (100)	4 (80)	
Fluoroquinolone	6 (50)	1 (14.3)	5 (100)	
Aminoglycoside	0 (0)	0 (0)	0 (0)	
Drug, generic name (at baseline)				
Clarithromycin	11 (91.7)	7 (100)	4 (80)	
Azithromycin	1 (8.3)	0 (0)	1 (20)	
Ethambutol	10 (83.3)	6 (85.7)	4 (80)	
Rifamycin	11 (91.7)	7 (100)	4 (80)	
Sitafloxacin	6 (50)	1 (14.3)	5 (100)	
Amikacin	0 (0)	0 (0)	0 (0)	

*ALIS* Amikacin liposome inhalation suspension, *GBT* Guideline-based therapy, *MAC Mycobacterium avium* complex

^a^interquartile range

### Patient Data Summary

Table 3 summarizes the clinical, radiological and microbial data of patients with MAC-PD after ALIS treatment initiation. All 7 patients in this culture conversion group achieved sputum culture conversion within the first 1–3 months. Susceptibility testing showed CLA-resistant isolates in 3 patients (#2, 7, 12). All had AMK-sensitive isolates, and all 5 isolates detected 6 months later remained AMK-susceptible.

**Table 3:** Serial changes in sputum examinations, microbiological, and clinical radiological data

Case number	Sputum culture conversion	Sex	Age (years)	Sputum examination results / Timeline														Total treatment duration before ALIS (months)	Baseline MIC of CLA (μg/mL)	6 months after MIC of CLA (μg/mL)	Baseline MIC of AMK (μg/mL)	6 months after MIC of AMK (μg/mL)	Radiological pattern
				Baseline						1-3 months				3-6 months									
				Count	Culture	PCR	Smear	Time to positive culture (days)	Smear Grade with Ziehl-Neelsen staining	Count	Culture	PCR	Smear	Count	Culture	PCR	Smear						
#1	＋	F	63	3	1	1	0	26		1	0	0	0	2	0	0	0	14	0.25		4		Non-cavitary NBE type
#2	－	M	73	2	2	1	2	21, 30, (median, 25.5)	±, 2+	2	2	2	2	2	2	2	2	69	>32	>32	8	8	FC type
#3	＋	F	73	3	1	1	0	42		1	0	0	0	2	0	0	0	21	0.5		16		Non-cavitary NBE type
#4	＋	F	70	2	1	1	1	32	±	1	0	0	0	3	0	0	0	17	0.06		2		Non-cavitary NBE type
#5	－	M	61	2	2	2	1	28, 22 (median, 25)	2+	2	2	2	2	2	2	1	1	32	0.5	0.5	8	8	Cavitary NBE type
#6	＋	F	65	3	1	1	1	35	±	1	0	0	0	2	0	0	0	26	0.125		0.5		Non-cavitary NBE type
#7	－	F	72	4	1	2	2	32	±, ±	1	1	1	1	3	2	2	1	96	>32	>32	2	8	Cavitary NBE type
#8	＋	F	52	3	2	2	1	25, 30 (median, 27.5)	±	2	0	0	0	3	0	0	0	24	0.25		4		Non-cavitary NBE type
#9	－	M	64	3	3	3	3	28, 32, 28 (median, 28)	2+, 2+, 1+	1	1	1	1	3	2	3	3	62	0.25	0.5	8	16	Cavitary NBE type
#10	＋	F	72	2	1	2	2	28	±, ±	1	0	0	0	2	0	0	0	9	2		16		Cavitary NBE type
#11	＋	F	81	4	1	4	0	51		3	0	1	0	2	0	1	0	29	0.125		4		Cavitary NBE type
#12	－	F	61	8	6	8	6	35, 23, 23, 35, 35, 26 (median, 34.5)	1+, 2+, ±, ±, 2+	3	3	3	2	1	1	1	1	25	>32	>32	4	4	Cavitary NBE type

*ALIS *Amikacin liposome inhalation suspension, *MIC *Minimum inhibitory concentration, *M *male, *F *Female, *NBE *Nodular bronchiectatic, *FC *Fibrocavitary

**Fig. 1:**
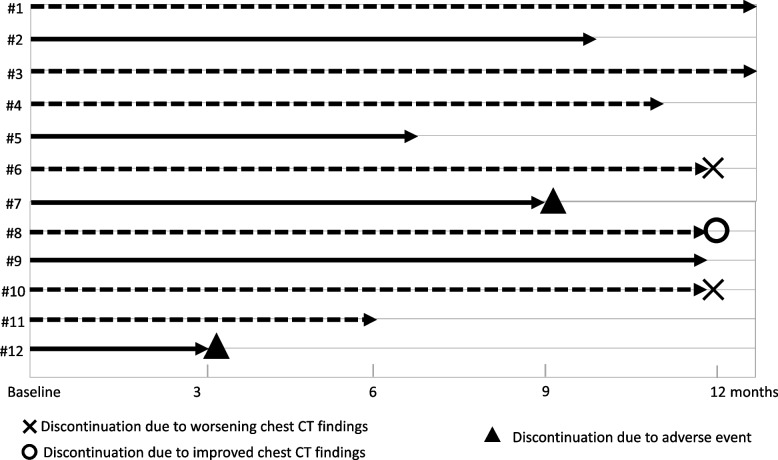
Treatment Persistence of Amikacin liposome inhalation suspension. Black dotted lines indicate patients with sputum culture conversion. Black lines indicate patients with non-culture conversion. × indicates discontinuation due to worsening chest CT findings. ◯ indicates discontinuation due to improved chest CT findings. △ = indicates discontinuation due to adverse event

Figure 1 shows ALIS treatment persistence status. Eleven patients completed the first 6 months of treatment. As at March 2023, 5 patients had discontinued: 2 patients (#7,12) due to adverse events, 2 patients (#6,10) due to worsening chest CT findings, and 1 patient (#8) due to favorable results.

### ALIS Adverse Event Profiling

Table 4 showed adverse events for ALIS. 11 (91.7%) patients had some adverse effects due to ALIS. Dysphonia was the most common, observed in 8 patients (66.7%); 2 within 2 weeks, 5 within 2–4 weeks, and 1 within 6–10 weeks. All patients improved with warm water gargle before and after ALIS inhalation. Also, with alternate-day ALIS administration, 7 patients resumed daily administration (1 patient continues to receive alternate-day ALIS). Other symptoms were cough in 3 patients (25%), pharyngula in 2 patients (16.7%), oral cavity discomfort in 1 patient (8.3%), and hemoptysis in 1 patient (8.3%) within the first 4 weeks. All patients subsequently improved. One patient (#12) discontinued at 3 months after ALIS initiation due to ototoxicity (Fig. 1). Another patient (#6) discontinued at 9 months after ALIS initiation due to hypersensitivity pneumonitis and ototoxicity.

**Table 4:** Adverse Event Profiling for ALIS

Adverse Event	n (%)
Dysphonia	8 (66.7)
Cough	3 (25)
Pharyngula	2 (16.7)
Oral discomfort	1 (8.3)
Hemoptysis	1 (8.3)
Hypersensitivity pneumonitis	1 (8.3)
Digestive symptoms	0 (0)
Vertigo	0 (0)
Nephrotoxicity	1 (8.3)^a^
Ototoxicity	2 (16.7)^b^

*ALIS *Amikacin liposome inhalation suspension

^a^9 months after treatment initiation

^b^1 of 2 patients 10 months after treatment initiation

### Serial changes in chest CT score, CAT score, and body weight

**Fig. 2:**
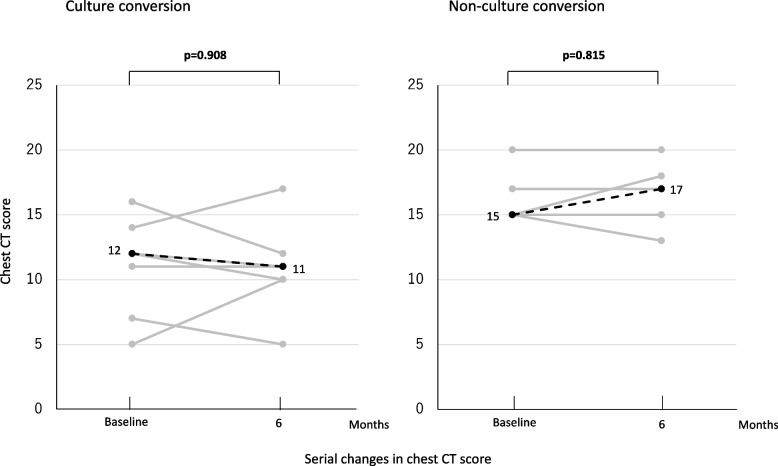
Serial changes in chest CT score. Gray lines indicate change in chest CT score in each patient after Amikacin liposome inhalation suspension initiation for *Mycobacterium avium* complex pulmonary disease. Black dotted lines indicate the median for all patients. A paired t-test was used to compare values at 6 months with baseline

Figure 2 shows serial changes in chest CT score in both groups. Median CT score at 6 months did not differ compared with baseline in both groups [(12 vs. 11; *p*=0.908) (15 vs. 17; *p*=0.815)]. In the culture conversion group, 4 of 7 patients showed improvement, 2 showed worsening, and the remaining 1 showed no change. In the non-culture conversion group, 1 of 5 patients showed improvement, 1 showed worsening, and the remaining 3 showed no change.

**Fig. 3:**
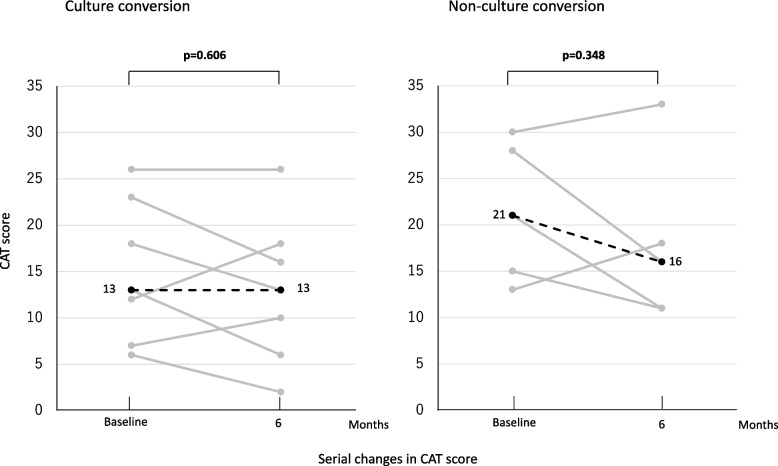
Serial changes in CAT score. Gray lines indicate change in CAT score in each patient after Amikacin liposome inhalation suspension initiation for *Mycobacterium avium* complex pulmonary disease. Black dotted lines indicate the median for all patients. A paired t-test was used to compare values at 6 months with baseline

Figure 3 shows serial changes in CAT score in both groups. Median CAT score at 6 months did not differ compared with baseline in both groups [(13 vs. 13; *p*=0.606) (21 vs. 16; *p*=0.348)]. The 3-point improvement the MCID in CAT score was achieved in 4/7 (57%) patients in the culture conversion group and in 3/5 (60%) in the non-conversion group.

**Fig. 4:**
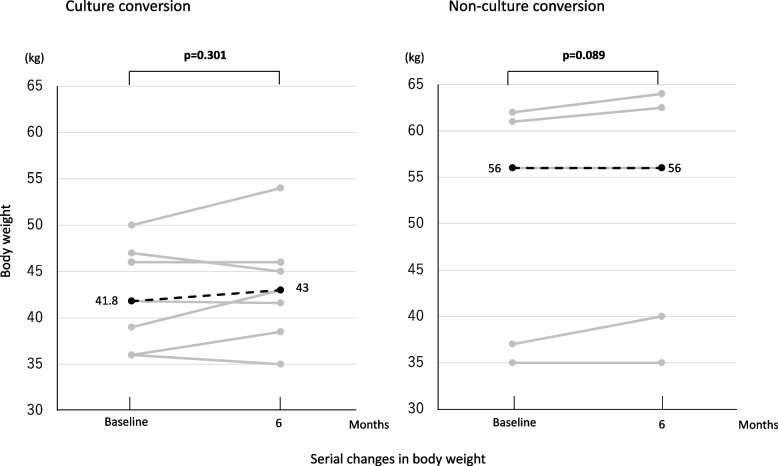
Serial changes in body weight (kg). Gray lines indicate change in body weight in each patient after initiation of Amikacin liposome inhalation suspension for *Mycobacterium avium* complex pulmonary disease. Black dotted lines indicate the median for all patients. A paired t-test was used to compare values at 6 months with baseline

Figure 4 shows serial changes in body weight in both groups. Median body weight at 6 months did not differ compared with baseline for both groups [(41.8 vs. 43 kg; *p*=0.301) (56 vs. 56 kg; *p*=0.089)]. In the culture conversion group, 3 of 7 patients had weight gain, with weight loss and no change in 2 patients each, respectively. In the non-culture conversion group, 3 patients gained weight with no change in the remaining.

## Discussion

In this study, 7 of 12 (58.3%) patients with refractory MAC-PD achieved sputum culture conversion with ALIS under GBT. However, the sputum negative conversion rate was unsatisfactory in patients with cavitary lesions, CLA-resistant isolates, and prolonged pre-ALIS initiation treatment duration. In the CONVERT study, 65 of 224 (29%) patients with refractory MAC-PD achieved sputum culture conversion at 6 months with ALIS treatment [3]. Subsequently, 24 of 90 (26.7%) patients with refractory MAC-PD and no prior ALIS exposure, and who did not achieve sputum culture conversion, achieved sputum culture conversion at 6 months of ALIS treatment [12]. The comparatively higher rates of sputum culture conversion in our study vs. these reports can be attributed to the inclusion of patients who were easier to treat than in the CONVERT study with shorter pre-ALIS treatment duration (2.1 vs. 4.5 years).

Contrarily, the proportion of CLA-resistant isolates was comparable to that of the CONVERT study [25% (3 of 12) vs. 22.9% (51 of 224)]. In the CONVERT study, sputum culture conversion was achieved by 7 of 51 (13.7%) and 58 of 173 (33.5%) patients with CLA-resistant and susceptible isolates, respectively. In this study, sputum culture conversion was achieved by 0 of 3 (0%) and 7 of 9 (77.8%) patients with CLA-resistant and susceptible isolates, respectively. This demonstrates that CLA sensitivity is also an important factor in the therapeutic efficacy of ALIS. All isolates in our study were AMK-sensitive, while in the CONVERT study 222/223 (99.6%) strains had an MIC of AMK < 64 μg/mL, so the effect of AMK-resistant isolates on treatment efficacy could not be examined. It has been reported that prior treatment with other AMK formulations can lead to AMK resistance [13]. In our study, 2 of 5 isolates showed elevated AMK MIC after 6 months of inhaled AMK, but none reached 128 μg/mL, the ALIS breakpoint proposed by the CLSI [7].

Further, the CONVERT study did not investigate differences in treatment efficacy by MAC-PD type. MAC-PD is comprehensively classified into nodular bronchiectatic (NBE) and fibrocavitary (FC) types, both known to affect treatment outcomes [14]. Another study reported poorer prognosis in MAC-PD patients with cavitary lesions regardless of type, than those without cavitary lesions [15]. Also, our study found lower sputum conversion rates for MAC-PD patients with cavitary lesions. Nevertheless, 1 of 5 patients in the non-conversion group with cavitary lesions showed improved chest CT findings including resolving cavitary lesions. Further, this patient also showed weight gain. Thus, sputum culture conversion alone may not correspond to the whole effectiveness of ALIS. Long-term ALIS treatment may be recommended for patients with improved chest CT findings, regardless of sputum culture conversion status. Conversely, 2 of 7 patients in the culture conversion group had worsened chest CT findings and weight loss. In these patients, MAC lesions were present in central airways predominantly which might be eradicated by ALIS inhalation. Thus, improved CT findings do not always correspond to sputum culture conversion. The fact that the use of ALIS achieved an MICD of CAT score in approximately 60% of cases, with or without sputum culture conversion, suggests that this may reflect a decrease in bacterial abundance due to ALIS with or without culture conversion.

Adverse events were observed in 11 of 12 (91.7%) patients in this study. Severe adverse events leading to ALIS treatment discontinuation were observed in 2 (16.7%) patients (hypersensitivity pneumonitis and ototoxicity). In the CONVERT study, 219 of 224 patients (98.2%) in the ALIS + GBT arm reported some form of adverse events; in 17.4% of patients this led to ALIS discontinuation. According to Morita et al., while 9 of 11 (81.8%) patients reported some adverse events, no serious adverse event was observed [16]. The proportion of these adverse events is very similar.

In our study, dysphonia was observed in 8 of 12 (66.7%) patients; 7 cases occurred within the first month. Dysphonia was the most reported adverse event in the CONVERT study, seen in 47% of patients [3] also noted in 73.1% (19/26) and 72.7% (8/11) from other reports [16, 17]. In this study, dysphonia improved with warm water gargle before and after ALIS inhalation for all patients. A previous study describes satisfactory management of dysphonia using lozenges, warm water or glycerin gargle, changing ALIS administration time to evening, and reducing or briefly interrupting dosing frequency [17].

Hypersensitivity pneumonitis was reported in 3.1% of patients in another CONVERT study [18]. It showed hypersensitivity pneumonitis with fever and dyspnea that developed 2 weeks after ALIS initiation, but imaging findings improved at 2 weeks post-withdrawal [19]. In this study, 1 patient had chest CT findings suggestive of hypersensitivity pneumonitis at 9 months after initiation. There were no added symptoms such as dyspnea, and chest CT findings subsequently improved at 3 months after withdrawal of ALIS.

In the CONVERT study, AMK-induced ototoxicity was observed in 17 of 223 (7.6%) patients with hearing loss in 10 of 223 (4.5%) [3]. In this study, 2 of 12 (16.7%) patients developed ototoxicity, an adverse event that should be monitored cautiously.

This study has limitations. As a single-center study in a small number of patients, the findings may not be generalizable to a larger, more diverse population. Also, the study duration was short, long-term follow up might further highlight potential issues with resistant isolates.

## Conclusion

ALIS facilitated sputum culture conversion within 6 months in 58.3% of patients with refractory MAC-PD. Sputum culture conversion was significantly more frequent in CLA-susceptible strains and in cases with fewer cavitary lesions. Early treatment with ALIS is desirable for patients with refractory MAC-PD receiving GBT. Improved CT findings after ALIS treatment did not always correspond to sputum culture conversion.

## Data Availability

All data generated or analyzed during this study are included in this article. Further enquiries can be directed to the corresponding author.

## Abbreviations

ATS: American Thoracic Society
ALIS: Amikacin liposome inhalation suspension
AMK: Amikacin
BMI: Body mass index
COPD: Chronic Obstructive Pulmonary Disease
CAT: Chronic Obstructive Pulmonary Disease assessment testing
CT: Computed tomography
CI: Confidence interval
CLA: Clarithromycin
CLSI: Clinical and Laboratory Standards Institute
ERS: European Respiratory Society
ESCMID: European Society of Clinical Microbiology
FC: Fibro cavitary
GPL: Glycopeptidolipid
HRQoL: Health-related quality of life
IDSA: Infectious Disease Society of America
IV: Intravenous
HRCT: High-resolution CT
MAC: *Mycobacterium**avium* Complex
MAC-PD: *Mycobacterium**avium* Complex pulmonary disease
MCID: Minimal clinically important difference
MIC: Minimum inhibitory concentration
NTM: *Nontuberculous mycobacteria*
NB: Nodular bronchiectasis
OR: Odds Ratio
SM: Streptomycin
